# Overexpression of the *WOX5* gene inhibits shoot development

**DOI:** 10.1080/15592324.2022.2050095

**Published:** 2022-04-01

**Authors:** Kyounghee Lee, Jin Hoon Won, Pil Joon Seo

**Affiliations:** a Department of Chemistry, Seoul National University, Seoul, Korea; b Research Institute of Basic Sciences, Seoul National University, Seoul, Korea; c Plant Genomics and Breeding Institute, Seoul National University, Seoul, Korea

**Keywords:** Stem cell niche, RAM, SAM, WOX5

## Abstract

WUSCHEL-RELATED HOMEOBOX 5 (WOX5) is a member of the WUSCHEL (WUS) homeodomain transcription factor family. *WOX5* is expressed mainly in the quiescent center (QC) and confers stem cell identity in the root apical meristem (RAM). Consistent with the role of *WUS* in repressing root meristem development, we found that ectopic expression of *WOX5* disrupted shoot development by repressing shoot-related genes, such as *YABBY1* (*YAB1*). Our findings suggest that WOX5 and WUS potentially confer different tissue identities and specify RAM and SAM, respectively.

Two primary meristems, shoot apical meristem (SAM) and root apical meristem (RAM), govern plant growth and development. Stem cell populations in the SAM and RAM are delicately modulated by a group of mitotically less-active cells comprising the organizing center (OC) and quiescent center (QC), respectively.^1,2^ Stem cell niches are established using a subset of WUSCHEL (WUS) family proteins. The *WUS* gene, which is specifically expressed in the OC, is responsible for SAM formation,^1^ whereas the QC-expressed *WUSCHEL-RELATED HOMEOBOX 5* (*WOX5*) gene plays a key role in RAM formation.^3^ Consistent with the origin of WUS and WOX5 proteins from a common ancestor,^4^ both of these transcription factors perform a similar function in maintaining stem cell pluripotency, as shown by the complementation of *wus* mutants by *pWUS::WOX5* expression or that of *wox5* mutants by *pWOX5::WUS*.^4^

In addition to their overlapping functions, WUS and WOX5 have also been suggested to perform distinct functions, especially in tissue identity establishment. For example, conditional overexpression of the *WUS* gene leads to shoot organogenesis even from root tissues, possibly by generating shoot stem cell niche and also by inhibiting root meristem regulators such as *PLETHORA 1* (*PLT1*).^5^ These observations suggest the possibility that the WOX5 transcription factor is also suspected to affect shoot development, in addition to its original role in the maintenance of root stem cells.^6^

To investigate the possibility that WOX5 could affect shoot development, we used transgenic *Arabidopsis* plants expressing the *35S::WOX5-GLUCOCORTICOID RECEPTOR* (*GR*) construct,^6^ which enables dexamethasone (DEX)-induced nuclear targeting of the WOX5-GR fusion protein (Figure 1a). While wild-type seedlings were insensitive to DEX treatment and showed normal growth and development, regardless of DEX application, the DEX-treated *35S::WOX5-GR* transgenic plants displayed dramatically disturbed shoot development (Figure 1b,c). In contrast, the ectopic expression of *PLT2*, which regulates root stem cell development similar to *WOX5*,^7^ in *35S::PLT2-GR* plants led to the production of normal shoots following DEX treatment unlike *35S::WOX5-GR*, although overall shoot size was reduced (Supplemental Figure S1). These data suggest that WOX5 can suppress the shoot developmental program.

**Figure 1.**
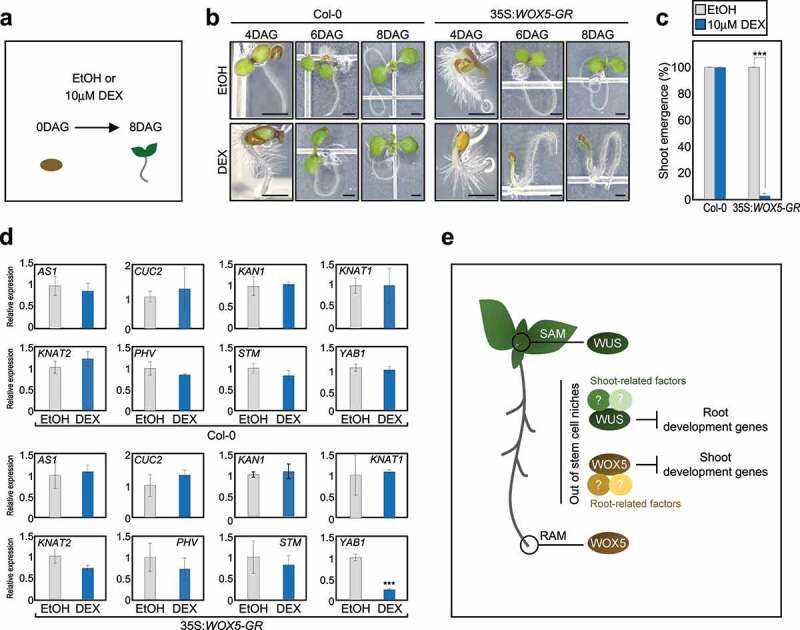
WOX5 inhibits shoot development. (a) Schematic representation of the study design. (b) Representative phenotype of dexamethasone (DEX)-treated *35S::WOX5-GR Arabidopsis* seedlings. Wild-type and *35S::WOX5-GR* plants were germinated on Murashige and Skoog (MS) medium supplemented with or without microM DEX, and grown under long-day (LD) conditions for 8 days. Scale bars = 1 mm. DAG, days after germination. (c) Percentage of shoot emergence. Cotyledon expansion was used as a phenotypic marker to evaluate shoot emergence. Statistically significant differences were determined using Student’s *t*-test (*n*= 33, ***P < 0.001). (d) Expression profiling of genes involved in SAM development. Total RNA was isolated from shoots of 11-day-old seedlings treated for 3 h with microM DEX or ethanol (EtOH). Transcript accumulation was analyzed by RT-qPCR. The *eIF4a* gene was used as an internal control. Data represent the mean ± standard error of the mean (SEM). Asterisks indicate statistically significant differences (***P < 0.001; Student’s *t*-test). (e) Schematic of WUS and WOX5 working model. OC-expressed WUS and QC-expressed WOX5 regulate the specification of SAM and RAM, respectively. In addition to their conserved function in pluripotency acquisition, WUS and WOX5 may also have potential roles in conferring distinct tissue identity. Ectopic activation of *WUS* inhibits root development genes even in roots and promotes shoot organogenesis from root tissues. In contrast, ectopic activation of *WOX5* inhibits shoot development genes in shoots possibly through distinct interacting proteins that define tissue identity. SAM, shoot apical meristem; RAM, root apical meristem.

To gain insight into the role of WOX5 in shoot development, we analyzed expression of several genes involved in shoot development, including *ASYMMETRIC LEAVES 1* (*AS1), CUP-SHAPED COTYLEDON 2* (*CUC2), KANADI 1* (*KAN1), KNOTTED-LIKE FROM ARABIDOPSIS THALIANA 1* (*KNAT1), KNAT2, PHAVOLUTA* (*PHV), SHOOT MERISTEMLESS* (*STM*), and *YABBY1* (*YAB1*),^8-10^ in shoots of wild-type and *35S::WOX5-GR* plants treated with DEX for 3 hours. Quantitative real-time RT-PCR (RT-qPCR) analysis revealed that DEX treatment specifically repressed *YAB1* expression in *35S::WOX5-GR*, but did not affect expression of other genes examined (Figure 1d). Given that YAB1 is a member of YABBY transcription factor family, which is specifically expressed in lateral organs of shoots,^11,12^ ectopic expression of *WOX5* facilitated the inhibition of shoot developmental genes out of shoot stem cell niche. The regulation of *YAB1* by WOX5 occurred possibly in SAM peripheral zone and abaxial domain of leaves, where *YAB1* gene is mainly expressed.^11,13,14^ We expect that WOX5 may further regulate a wide spectrum of shoot-expressed genes, in addition to *YAB1*, which should be examined in the future.

*WOX5* and *WUS*, which originated from a common ancestor, exhibit conserved functions in the SAM and RAM, specifically during maintenance of stem cell niches in these meristematic tissues.^4^ Complementation of *wus-1* by *WOX5* expression driven by the native *WUS* promoter corrects the defect in SAM development.^4^ Similarly, *pWOX5::WUS* expression rescues abnormal RAM development in *wox5* mutants.^4^ While the overlapping functions of WUS and WOX5 are reflected in stem cell niches, they also potentially have unique functions. Ectopic expression of *WUS* inhibits root development possibly via suppression of root stem cell regulators.^5^ Our results also showed that ectopic expression of *WOX5* inhibits shoot development probably by repressing shoot-related genes. Since WUS and WOX5 complement functions each other in stem cell niches,^4^ their opposite functions in tissue identity establishment are most likely facilitated in the regions outside of stem cell niches, which might be related to the maintenance of body axis. Outside of stem cell niches, WUS and WOX5 may have more chance to interact with shoot-related and root-related proteins, respectively, which repress opposite tissue identity. Indeed, the unique protein interactome of each protein was suspected,^15^ and a list of interacting proteins of WOX5 and WUS were related to tissue-specific factors (Supplemental Figure S2), suggesting that spatial mis-expansion may lead to new regulatory repertoires via distinct molecular interactions.^16^ We could raise the possibility that the spatial mis-expression caused biological artifacts. Nonetheless, the genetic impact needs to be understood and can sometimes also be applied to artificial control of plant development. Overall, we propose that, while WUS and WOX5 exhibit a conserved function owing to their common ancestor, their subsequent functional divergence might have led to evolution of their tissue-specific roles in the SAM and RAM, respectively.

## Materials and methods

### Plant materials and growth conditions

*Arabidopsis thaliana* ecotype Columbia (Col-0) was used in all experiments, unless specified otherwise. Plants were grown at 22–23°C under long-day (LD) conditions (16 h light/8 h dark) using white fluorescent lamps (150 μmol photons m^−2^s^−1^). For DEX treatment, *35S::WOX5-GR*^6^ and *35S::PLT2-GR*^17^ seeds were germinated in Murashige and Skoog (MS) medium supplemented with or without 10 μM DEX.

### RT-qPCR analysis

Total RNA was extracted from the plant materials of interest using the TRI Reagent (Takara Bio), according to the manufacturer’s instructions. First-strand cDNA was synthesized from 2 μg of total RNA using Moloney Murine Leukemia Virus reverse transcriptase (Dr. Protein) and dT20 oligos. The cDNA was diluted to a volume of 100 μl with Tris-EDTA (TE) buffer, and 1 μl of the diluted cDNA was used for RT-qPCR.

The RT-qPCR reactions were performed on 96-well plates using the StepOnePlus Real-Time PCR System (Applied Biosystems). Gene expression levels were normalized relative to that of *EUKARYOTIC TRANSLATION INITIATION FACTOR 4A1* (*eIF4A*; At3g13920). The primers used for RT-qPCR are listed in Supplemental Table S1. The relative gene expression levels were quantified using the comparative ΔΔC_t_ method. The threshold cycle (C_t_) for each reaction was determined automatically by the analysis software using default parameters (Applied Biosystems). The specificity of RT-qPCR reactions was determined by melting curve analysis.

## Supplementary Material

Supplemental Material
