# Supplementary material for: Overexpression of the WOX5 gene inhibits shoot development
Source: Plant Signal Behav. 2022 Apr 1;17(1):2050095. doi: 10.1080/15592324.2022.2050095 (PMC8986225; doi:10.1080/15592324.2022.2050095)
Supplement: Supplemental Material [file KPSB_A_2050095_SM0121.pdf]

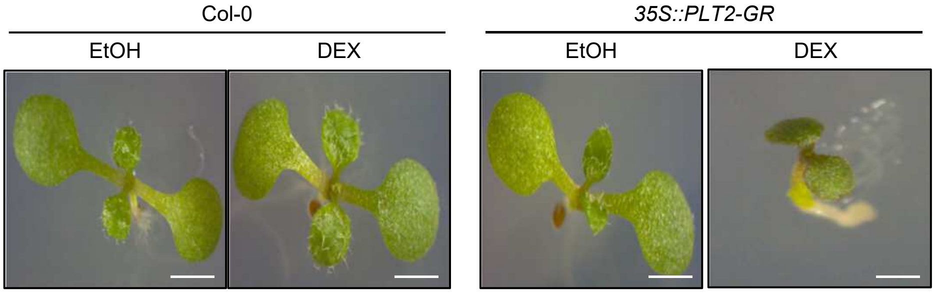


**Supplemental Figure S1. Representative phenotype of wild-type and *35S::PLT2-GR*.**
Wild-type and *35S::PLT2-GR* plants were germinated from Murashige and Skoog (MS) medium supplemented with 10 μM dexamethasone (DEX) and were grown under long-day (LD) conditions for 7 days. Scale bars = 1 mm.

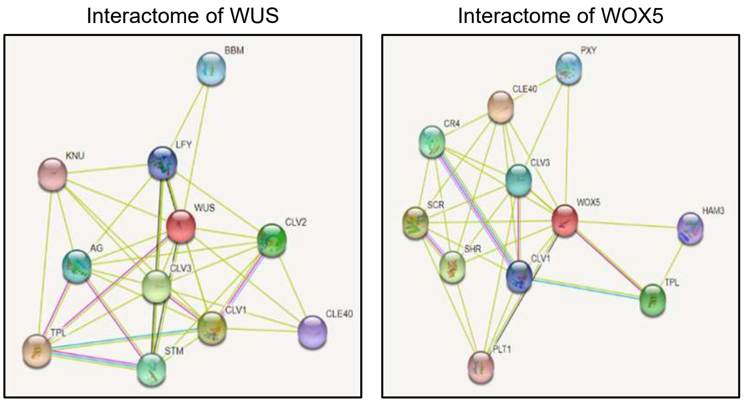


**Supplemental Figure S2. Protein interactome of WUS and WOX5.**
Interacting proteins of WUS and WOX5 transcription factors were obtained from STRING database. Nodes represent proteins and edges represent potential protein-protein interactions. BBM, BABY BOOM; LFY, LEAFY; KNU, KNUCKLES; WUS, WUSCHEL; AG, AGAMOUS; CLV1, CLAVATA1; CLV2, CLAVATA2; CLV3, CLAVATA3; TPL, TOPLESS; STM, SHOOTLESS; CLE40, CLAVATA3/ESR-RELATED40; PXY, PHLOEM INTERCALATED WITH XYLEM; CR4, CRINKLY4; SCR, SCARECROW; SHR, SHORT ROOT; WOX5, WUSCHEL RELATED HOMEOBOX5; HAM3, HAIRY MERISTEM3; PLT1, PLETHORA1.

| Primer | Usage | Sequence | |
|---|---|---|---|
| | | F | R |
| eIF4a | RT-qPCR | TGACCACACAGTCTCTGCAA | ACCAGGGAGACTTGTTGGAC |
| AS1 | RT-qPCR | ATTGACGAGAGTAAGTACGA | CTGAACTTGTTGTTCAGAAT |
| CUC2 | RT-qPCR | CTGGATCCGACCACTACCCT | GGACACGTGCTCGGTTATTG |
| KAN1 | RT-qPCR | AACAAGCCTGCTGCTTCATC | CGTTTCCATTTATGCCCATT |
| KNAT1 | RT-qPCR | TTACTCCATGTTAATGAGAGCCA | ACCAACATGTCACAGTATGCTTC |
| KNAT2 | RT-qPCR | CGAGAGAACCATGTGTACAAGAG | CTTCTCTCAGTTCCTCGTCAGAT |
| PHV | RT-qPCR | TTGGTTCCAGAATCGCAGA | CACTGTCTGAAGACGAGCTGA |
| STM | RT-qPCR | AACAATAATGGGTCATC | TTCCTTTCTTCCTCTTCTTC |
| YAB1 | RT-qPCR | CCAATCTCCTTTCGGTGAAC | GCATCTCTCAGCTCCTCCAG |

**Supplemental Table S1. Primers used in this study.**
The sizes of PCR products ranged from 80 to 300 nucleotides in length. F, forward primer; R, reverse primer.
